# Supplementary material for: Chemical Characterization and Seasonality of Ambient Particles (PM2.5) in the City Centre of Addis Ababa
Source: Int J Environ Res Public Health. 2020 Sep 24;17(19):6998. doi: 10.3390/ijerph17196998 (PMC7579520; doi:10.3390/ijerph17196998)
Supplement: Supplementary file 1 [file ijerph-17-06998-s001.pdf]

Supplementary Information

S1(a) S1(b)

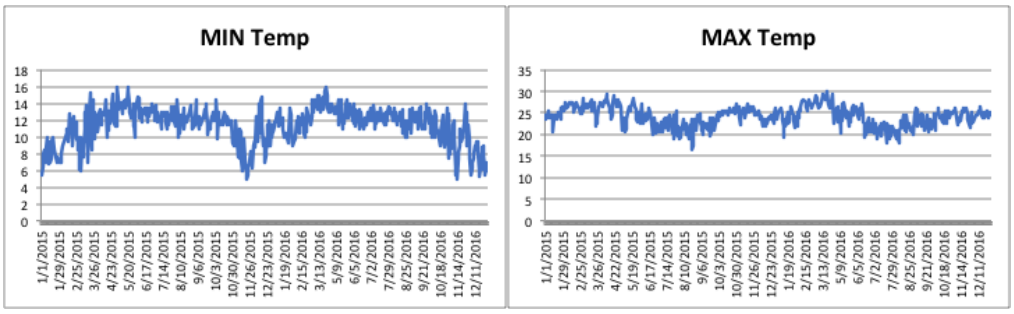

S1(C)

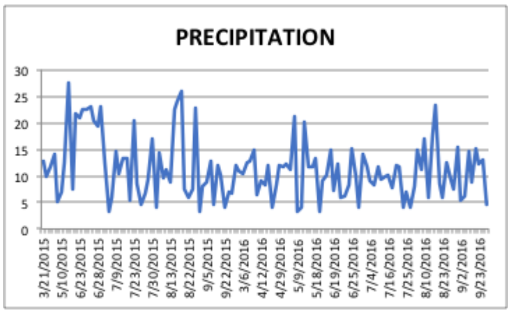

Supplemental Figure S1. Mean daily Minimum Temperature (^{0}C) (a), Maximum Temperature (^{0}C) (b) and Precipitation (mm) (C) in Central Addis Ababa during 2015-2016.

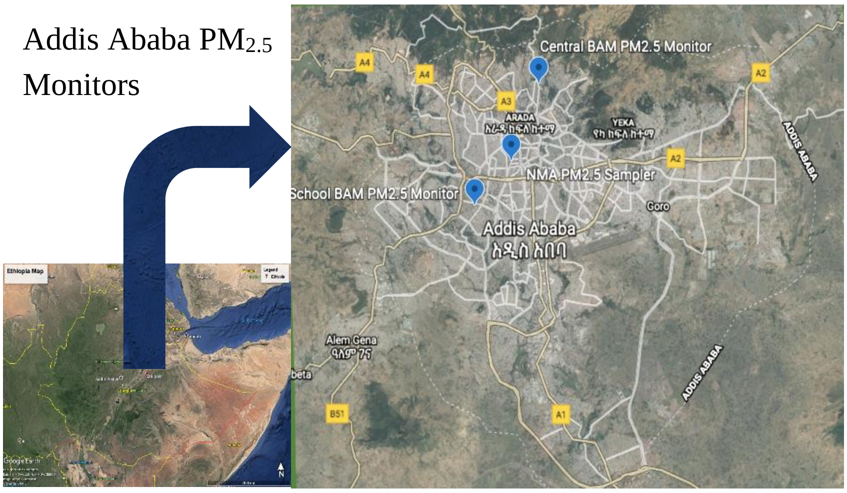

Supplemental Figure S2. Fine PM Monitoring stations in Addis Ababa, Ethiopia. NMA $PM_{2.5}$ Sampler (Met Station site - this study); US Diplomatic Post BAM Monitors (Central and School sites) are shown in figure with blue pin.

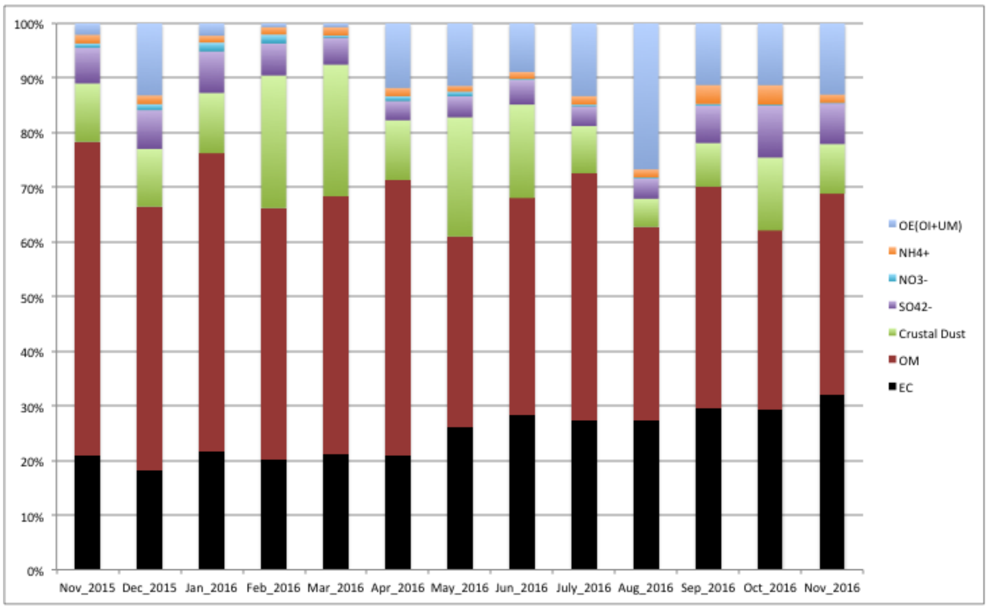

Supplementary Figure S3. The percentage contribution of major mass constituents of $PM_{2.5}$ in Central Addis Ababa; EC=Elemental carbon. OM=Organic matter; Other Ions= ws Na+, ws Cl-, ws K+, ws Ca^{2+}; UM= Unidentified matter.

Supplemental Table S1

Table S1. Crustal Enrichment Factor(CEF>10) of 15 trace-elements identified from $PM_{2.5.}$ samples in central Addis Ababa.

Element	[a]Mean CEF	STDEV CEF	MIN CEF	MAX CEF
Thallium (Tl)	11	10	1	21
Nickel (Ni)	14	14	0	28
Phosphorous (P)	15	84	0	99
Vanadium (V)	21	17	4	38
Molybdenum (Mo)	37	48	0	85
Boron (B)	69	92	0	161
Zinc (Zn)	87	72	15	159
Copper (Cu)	**115**	675	0	790
Arsenic (As)	**121**	124	0	245
Lead (Pb)	**124**	191	0	315
Tin (Sn)	**193**	909	0	1102
Sulfur (S)	**245**	174	71	419
Silver (Ag)	**648**	1905	0	2553
Antimony (Sb)	**3796**	11347	0	15143
Selenium (Se)	**19180**	40393	0	59573

[a] CEF>100 are listed in bold, showing significant PM toxic metal contamination from anthropogenic sources.
